# Functional Mobility in Individuals With Lower Limb Amputation: An Observational Study

**DOI:** 10.7759/cureus.52759

**Published:** 2024-01-22

**Authors:** Neha Mukkamala, Shivani Vala

**Affiliations:** 1 Physiotherapy, College of Physiotherapy, Sumandeep Vidyapeeth Deemed to be University, Vadodara, IND

**Keywords:** independence, prosthesis, functional mobility, lower limb amputation, timed up and go test

## Abstract

Introduction: Amputation leads to a permanent disability and brings a dramatic change in the life and function of the individual, more so in individuals with lower limb amputation. A lower limb amputation reduces mobility and can make persons dependent on assistive devices like crutches or a wheelchair. Restoring mobility and optimal physical functioning of an individual with lower limb amputation is the most important rehabilitation goal. There are very few studies that have quantified mobility deficits with valid outcome measures, especially in the Indian population. Our study aims to quantify the mobility deficit in individuals with lower limb amputation and add to the scant literature available on mobility values in the Indian population.

Methods: This was a cross-sectional study. Individuals with lower limb amputation who attended an orthotic and prosthetic clinic in Vadodara city were recruited for the study. Those individuals who were above 18 years of age and had undergone either unilateral or bilateral amputation, at least six weeks prior to assessment, were included in the study. Those individuals who had total impairment of vision and hearing, cognitive impairment, upper limb amputation, and ankle and foot amputation were excluded from the study. Functional mobility was assessed with the prosthesis worn, using the Timed “Up and Go” (TUG) test.

Results: There was a total of 54 individuals with lower limb amputation, 47 males and seven females. The mean age was 47.38±18.83 years. Transtibial (66.67%) was the most common amputation followed by transfemoral (27.8%). The mean TUG score for the total population was 20.19 ± 11.95 sec, for unilateral transfemoral amputation 20.26 ± 12.06 sec, and for unilateral transtibial amputation 20.01 ± 12.31 sec. There was a statistically significant direct relation of the TUG score with age (p=0.02), level of amputation (p<0.01), and length of time prosthesis was used (in years) (p=0.02) and a statistically significant inverse relation of TUG score with the cause of amputation (traumatic, p=0.02, non-traumatic, p=0.03), assistive devices used for mobility (p<0.01), and number of hours the prosthesis was worn in a day (p<0.01). There was a significant negative correlation between the duration of amputation and TUG score (r=-0.282, p<0.05)

Conclusion: The functional mobility was reduced in individuals with lower limb amputation. There was a statistically significant direct relation of functional mobility with age, cause of amputation, level of amputation, and length of time of prosthesis used, and a statistically significant inverse relation with the number of hours of use of prosthesis in a day and assistive devices used. Individuals who were old, had a non-traumatic amputation, a higher level of amputation, those wearing a prosthesis for a short duration since amputation, who wore the prosthesis for a shorter duration during the day, and who used assistive devices for ambulation in addition to a prosthesis had longer TUG times. As the duration of amputation increased, the time taken for TUG decreased.

## Introduction

Amputation leads to a permanent disability and brings a dramatic change in the life and function of the individual, more so in individuals with lower limb amputation [1,2]. The limitations in the structure and function of the body due to amputation can impact a person's activity level as well as their ability to participate in society [1].

Mobility, a key component of independent living, enables the performance of activities of daily living. It provides independence and a higher quality of life [3]. A lower limb amputation reduces mobility and can make people dependent on assistive devices like crutches or a wheelchair. Reduced mobility due to amputation is influenced by age, level and cause of amputation, comorbidities, and physical fitness [4]. Restoring mobility and optimal physical functioning of an individual with lower limb amputation is the most important rehabilitation goal [1,2]. A prosthetic device can help people with a lower limb amputation to regain mobility and independence in daily living [5].

There are very few studies that have quantified mobility deficits with valid outcome measures, especially in the Indian population. Our study aims to quantify the mobility deficit in individuals with lower limb amputation and add to the scant literature available on mobility values in the Indian population.

## Materials and methods

This was a cross-sectional study that was approved by the Institutional Ethics Committee (IEC). The study was registered with Clinical Trials Registry India (CTRI No: CTRI/2022/09/045557). Individuals with lower limb amputation who attended an orthotic and prosthetic clinic in Vadodara city from June 2022 to February 2023 were recruited for the study. The study included individuals with unilateral or bilateral lower limb amputation, who were above 18 years of age, and individuals who had undergone amputation at least six weeks prior to assessment. Those individuals who had total impairment of vision and hearing, cognitive impairment, upper limb amputation, and ankle and foot amputation were excluded from the study. Informed consent was obtained from all those who were willing to participate in the study. A patient information sheet, which included the details of the study, was given to all those who took part in the study. A detailed assessment of all the individuals including, demographic information, education level, employment status, level and cause of amputation, time since amputation, medical comorbidities, assistive devices used for mobility and prosthesis use, was taken.

Outcome measure

The functional mobility of individuals with amputation was assessed using the Timed “Up and Go” (TUG) test. The TUG test was taken for the individuals with the prosthesis as follows: at the start of the test, the individual was seated on a chair with back support and arm rest. Then on command, the individual was asked to stand up from the chair, walk to a line 3m away, take a turn, walk back to the chair, and sit down. The individual was instructed to walk comfortably at a normal walking pace [6]. If the individual used any assistive devices in addition to the prosthesis, then the TUG was assessed along with the assistive device.

Sample size calculation

n’=  *NZ^2^P (1-P) *

 d^2^(N-1) + Z^2^P(1-P)

n’= Sample size with finite population correction; N=100 (Population size); Z=1.96 (Z statistic for a level of confidence); P=0.08 (Expected proportion); d=0.05 (Precision). Thus, the sample size was calculated as 54.

Statistical analysis

Statistical analysis was done using IBM SPSS Statistics for Windows, Version 25 (Released 2017; IBM Corp., Armonk, New York, United States). Descriptive statistics like mean and standard deviation was performed for background variables and amputation characteristics. The level of significance was set at p <0.05. Univariate regression analysis was used to see the association between TUG scores and individual variables. Pearson’s correlation coefficient was used to see the correlation between the duration of amputation and TUG score.

## Results

A total of 54 individuals with lower limb amputation were included in the study. The demographic data of 54 individuals are as follows (Table 1). Males constituted 87% of the population. The mean age for all the individuals was 47.38±18.83 years (range 18-84 years). The mean BMI was 24.96±4.61 kg/m^2^ (Table 1). Approximately 60% of the individuals had completed their graduation. The majority of the population was either employed or retired. The most common level of amputation was unilateral transtibial amputation (66.67%). The cause of amputation for the majority of the participants was traumatic (57.40%); 92.5% of the individuals with amputations wore a prosthesis. Seventy-eight percent of these individuals ambulated with a prosthesis only, without any assistive devices.

**Table 1:** Demographic data of the population

Variables	N
Age (years)	
18-30	15 (3 females, 12 males)
31-50	15 (2 females, 13 males)
51-70	17 (2 females, 15 males)
71-90	07 (all males)
Gender	
Males	47
Females	07
Level of education	
<10^th^ grade	07
>10^th^ grade	15
Graduate	32
Employment status	
Employed	24
Unemployed	12
Retired	18
Cause of amputation	
Congenital	03
Traumatic	31
Non traumatic	20
Level of Amputation	
Hip disarticulation	01
Transfemoral	14
Knee disarticulation	03
Transtibial	36
Type of amputation	
Unilateral	51
Bilateral	03
Assistive devices for mobility	
None	39
Cane	07
Walker	07
Axillary crutches	01

The mean scores of the TUG test according to different levels of amputation are given in Table 2. It shows that the individuals with a transfemoral amputation had a slightly higher mean and range of TUG scores than those with transtibial amputation. Hip disarticulation and knee disarticulation were included in the transfemoral group.

**Table 2:** Mean scores of the Timed “Up and Go” (TUG) test

	Mean TUG score±SD(sec)	Range(sec)
Total Population (n=54)	20.19 ± 11.95	7.20 -72
Transfemoral Amputation (unilateral) (n=13)	20.26 ± 12.06	17-72
Transtibial Amputation (unilateral) (n=38)	20.01 ± 12.31	7.2-52

Thirty-three percent with transtibial amputations and 78.6% with transfemoral amputations had TUG values greater than 19 sec. The mean scores of the TUG test in different age groups are given in Table 3. Individuals with a transfemoral level had higher TUG times than the transtibial level in any age group. Hip disarticulation and knee disarticulation were included in the transfemoral group.

**Table 3:** Mean scores of the TUG test in different age groups TUG: Timed "Up and Go"

Age group (years)	Level of amputation (unilateral)	Mean TUG score ± SD (sec)	Range (sec)
18-30	Transfemoral and transtibial (n=15)	16.83±8.64	9-43
	Transfemoral (n=5)	19.20±2.83	17.05-22.87
	Transtibial (n=10)	15.65±10.39	9-43
31-50	Transfemoral and transtibial (n=13)	17.49±7.52	7.2-31
	Transfemoral (n=4)	21.59±7.62	12.37-31
	Transtibial (n=9)	15.67±7.13	7.2-30
51-70	Transfemoral and transtibial (n=16)	20.93±11.01	9-43
	Transfemoral (n=5)	31.66±10.50	18.3-43
	Transtibial (n=11)	16.05±7.35	9-32.77
71-90	Transfemoral and transtibial (n=7)	31.27±21.92	13.97-72
	Transfemoral (n=1)	72	72
	Transtibial (n=6)	24.48±13.76	13.97-52

Association between TUG scores and individual variables was seen using univariate regression analysis (Table 4). Age, cause of amputation, level of amputation, length of time prosthesis was used since amputation, number of hours the prosthesis was worn in a day, and assistive devices used for mobility showed a significant association with the TUG score (Table 4). Thus, individuals who were old, with a non-traumatic amputation, with a transfemoral level of amputation, those who wore a prosthesis for a shorter duration since amputation and for a shorter duration in a day, and those who used assistive devices for ambulation in addition to a prosthesis had longer TUG times. There was a significant negative correlation between the duration of amputation and TUG score (r=-0.282, p<0.05).

**Table 4:** Association between the TUG score and individual variables using univariate regression analysis TUG: Timed "Up and Go"

Variable	Univariate regression for TUG		
	Coefficient	SE	p-value
Gender	3.91	4.86	0.42
Age (years)	0.19	0.08	0.02
Employment Status	3.46	3.29	0.30
Marital Status	4.42	3.43	0.20
Body Mass Index (BMI)	0.26	0.36	0.47
Cause of amputation: Traumatic	-7.45	3.16	0.02
Cause of amputation: Non-traumatic	7.22	3.25	0.03
Level of Amputation	8.82	3.26	<0.01
Assistive devices for mobility	-14.56	3.06	<0.01
Length of time prosthesis used (number of years)	13.84	5.97	0.02
Use of prosthesis in a day (hours)	-1.58	0.30	<0.01

## Discussion

The present study assessed the functional mobility using TUG in individuals with lower limb amputations. The study supports the finding that trauma is the main cause of amputation in developing countries with more than half of the individuals having a traumatic cause for amputation [1,2,7]. The majority of the individuals in the present study had a transtibial level of amputation, followed by transfemoral, which is in agreement with the literature [1,2]. Surgeons try and salvage as much of the limb as possible while operating so that the individuals are mobile and independent [2].

TUG was used in our study to assess functional mobility as it has demonstrated good inter-rater and intra-rater reliability, validity, and responsiveness [3,6,8]. It requires little space, time, or cost to administer and uses equipment that is readily available in the clinical setting. It also evaluates tasks performed frequently, like sitting to stand, turning while walking, and stepping in different directions and participants can walk at their preferred walking speed and with their walking aid [9]. In our study, the average values of TUG were high compared to Newton’s study [8], which reported TUG values of 9.2 ± 2.6 sec for transtibial and 12.1 ± 3.8 sec for the transfemoral level of amputation [8]. The TUG values in different age groups were also higher in our study compared to those in the study by Newton et al. (age groups: 40-49, 50-59, 60-69) [8]. However, the study by Newton et al. [8] had a smaller sample size of 37 participants, with very few participants in all the age groups. So conclusions from such a small sample cannot be deduced. Spaan and colleagues [5] and Christiansen et al. [10] reported average TUG times of 17.3±9.1 sec and 18.6±13.9 sec, which were lower than the values in our study. The average values in the study by Schoppen et al. (transtibial-23.8 (23.0) sec and transfemoral-28.3 (12.2) sec), done on 32 elderly individuals with unilateral amputations, were higher than our study [6]. TUG values of 19 sec or more, at six months post-discharge, have been reported to increase the risk of multiple falls in patients with unilateral lower limb amputation [9]. In our study, the majority with transfemoral amputations and one-third with transtibial amputations had TUG values greater than 19 sec. The reasons for increased time on the TUG at the transtibial level were ambulation without a prosthesis, but, with an assistive device and use of an assistive device in addition to a prosthesis to ambulate. The reasons for increased time on the TUG at the transfemoral level were ambulation with an assistive device and a shorter duration of amputation.

Age, level of amputation, and time since amputation influence the performance of mobility outcomes [4,8]. In the present study, we found a statistically significant direct relation between the age and TUG score, which is supported by other studies [4,8]. Increased age is associated with a higher number of comorbidities that may affect balance and mobility, in general, and walking with a prosthesis, in particular. Increased age is also associated with lesser use of prostheses [3,5,8]. Seth et al. reported that for each year of age, there was a corresponding 0.23 sec increase in TUG time [4]. Newton and colleagues found a significant association with age in the multivariate analysis [8].

Our study found a statistically significant direct relation between the level of amputation and TUG score, with higher levels of amputation being associated with longer TUG scores. This finding is in agreement with other studies [5,6,8,11]. Newton and Spaan and colleagues reported that the longer TUG times may be due to a poorer standing balance, asymmetry of gait, muscle imbalance due to the removal of the knee joint, and an increased energy expenditure associated with mobility/prosthesis [5,8,11]. It has also been reported that individuals with unilateral transtibial amputations had better walking distance and gait speed than transfemoral amputation [12].

As reported by Silva et al., lower-limb prosthesis plays a pivotal role in improving physical capacity and body image and carrying out activities of daily living (ADL) independently [13]. It is preferred among assistive devices as individuals can carry out their daily activities as naturally as possible and it helps to improve their self-esteem [14]. Prosthesis leads to increased independence in walking over time. The use of a prosthesis has been reported to be one of the most important factors influencing the physical health component of QOL [1]. We found a statistically significant direct relation between the number of years the prosthesis was worn and TUG, which is supported by literature [8]. Newton and colleagues reported the length of time each participant used their prosthesis to be the strongest predictor of performance on mobility tests like TUG and the 2-minute walk test. The longer the time since the prosthesis was worn, the longer the individual had time for physical and psychological adjustment, practice, and accommodation to the prosthesis and the amputation [8]. A study reported that the use of prostheses positively impacts the mobility of elderly participants. Time is an important factor in improving prosthesis use for gait and ADL [13].

We found a statistically significant negative correlation between the duration of amputation and TUG score, signifying that as the duration of amputation increased, the time taken for TUG decreased. This finding is supported by the study by Seth et al. [4], who found a linear association between the two variables.

Our study found a statistically significant direct relation between the cause of amputation (non-traumatic) with TUG scores, which is in contrast to results reported by Spaan et al. [5]. All individuals with non-traumatic amputation in our study were elderly. As age increases, the TUG score also increases [5].

Our study also found a statistically significant inverse relation between assistive devices used for mobility with TUG scores. Almost all the individuals who walked with an assistive device in addition to a prosthesis were elderly and the majority of them had a duration of amputation of less than one year. Thus, they may have had inadequate training for walking with the prosthesis, in turn affecting their walking ability. Newton et al. have reported that as individuals get older, they tend to have more comorbid conditions, which can impact their movement and balance, hence requiring an additional assistive device for mobility [8].

Limitations of the study

The limitations of the study were that individuals were recruited from only one orthotic and prosthetic clinic in the study and a healthy control group was not included for comparison of TUG values. 

## Conclusions

The functional mobility was reduced in individuals with lower limb amputation. There was a statistically significant direct relation of functional mobility with age, cause of amputation, level of amputation, and length of time of prosthesis was used, and a statistically significant inverse relation with the number of hours of use of prosthesis in a day and assistive devices used. Individuals who were old, had a non-traumatic amputation, higher level of amputation, who wore a prosthesis for a short duration since amputation, who wore the prosthesis for a shorter duration during the day, and who used assistive devices for ambulation in addition to a prosthesis had longer TUG times. As the duration of amputation increased, the time taken for TUG decreased.
